# Supplementary material for: The interactome of intact mitochondria by cross-linking mass spectrometry provides evidence for coexisting respiratory supercomplexes
Source: Mol Cell Proteomics. 2017 Dec 8;17(2):216–32. doi: 10.1074/mcp.RA117.000470 (PMC5795388; doi:10.1074/mcp.RA117.000470)
Supplement: Supplemental Data [file supp_RA117.000470_133922_0_supp_23978_fzffwf.zip › spectra_annotation/mito_spectra_annotation/16-1-5-1-4-1.pdf]

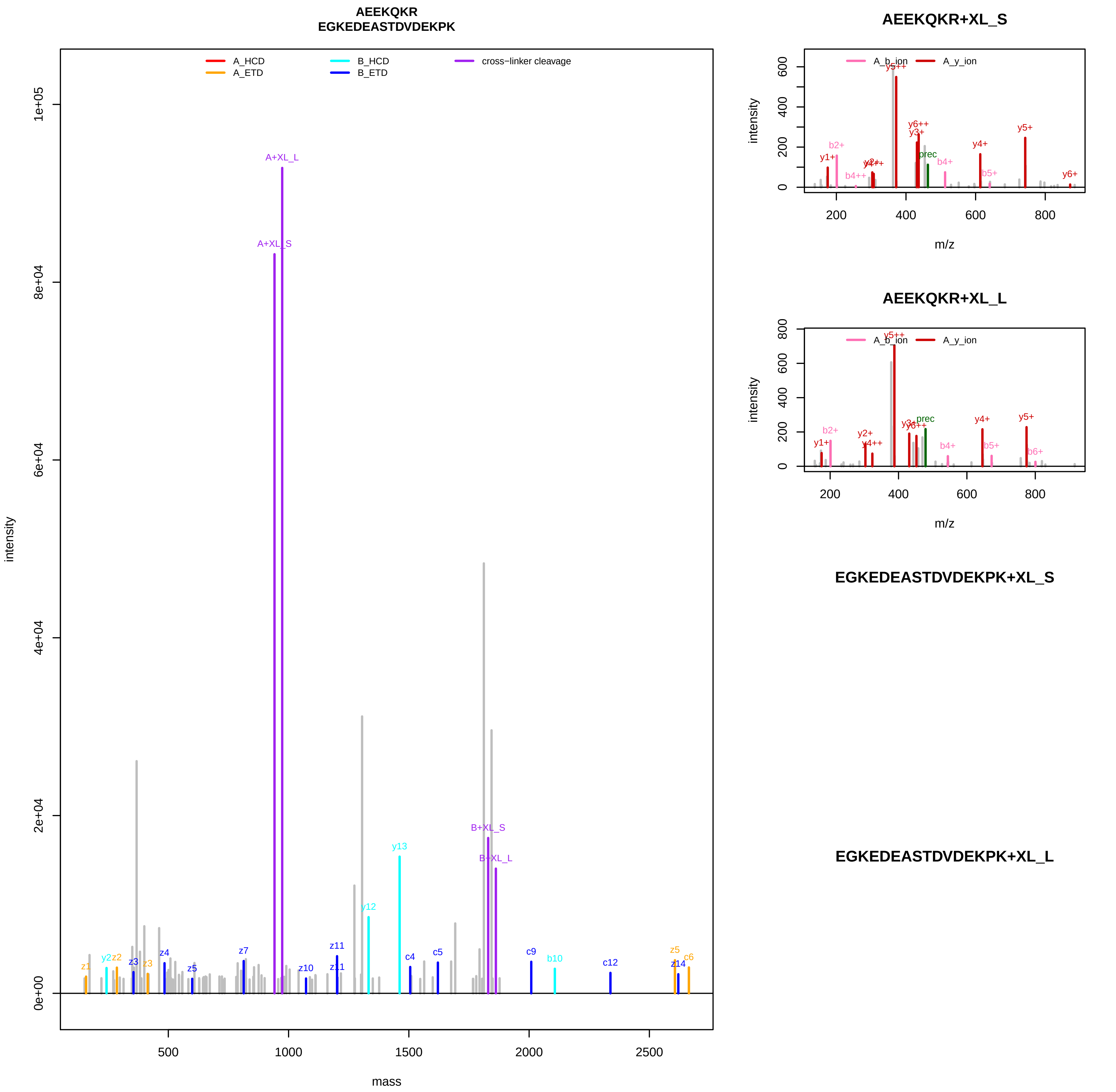
AEEKQKR
EGKEDEASTDVDEKPK
A_HCD
A_ETD
B_HCD
B_ETD
cross−linker cleavage
A+XL_L
A+XL_S
B+XL_S
B+XL_L
y13
y12
z11
z7
z4
z10
c4
c5
c9
b10
c12
z5
z14
c6
z1
y2
z2
z3
intensity
mass
0e+00
2e+04
4e+04
6e+04
8e+04
1e+05
500
1000
1500
2000
2500
AEEKQKR+XL_S
A_b_ion
A_y_ion
y5++
y6++
y3+
prec
b2+
y1+
b4++
y2+
y4++
b4+
y4+
y5+
b5+
y6+
m/z
0
200
400
600
800
AEEKQKR+XL_L
b6+
EGKEDEASTDVDEKPK+XL_S
EGKEDEASTDVDEKPK+XL_L
